# Beyond proofreading: POLD1 mutations as dynamic orchestrators of genomic instability and immune evasion in cancer

**DOI:** 10.3389/fimmu.2025.1600233

**Published:** 2025-06-30

**Authors:** Huiqing Chen, Jiayu Wei, Qi Tang, Guohui Li, Yajing Zhou, Zhen Zhu

**Affiliations:** ^1^ School of Life Sciences, Jiangsu University, Zhenjiang, China; ^2^ Department of Implantology and Prosthodontics, Zhenjiang Stomatological Hospital, Zhenjiang, China

**Keywords:** DNA polymerase delta, POLD1 mutation, cancer, genomic instability, immunotherapy

## Abstract

DNA polymerase delta (Pol δ) is a cornerstone of genomic stability, orchestrating DNA replication and repair through its catalytic subunit, POLD1. This subunit’s 3’–5’ exonuclease domain proofreads replication errors, ensuring fidelity. However, POLD1 mutations—particularly in this domain—disrupt this function, triggering genomic instability and a hypermutated state in cancers. This review delves into the multifaceted roles of POLD1 mutations, spotlighting their contributions to tumorigenesis and immunotherapy responses. Beyond their established link to syndromes like polymerase proofreading-associated polyposis (PPAP), these mutations unexpectedly enhance tumor immunogenicity in microsatellite-stable (MSS) tumors, previously considered largely resistant to immune checkpoint inhibitors (ICIs). By elevating tumor mutation burden and generating unique mutational signatures (e.g., SBS10d), POLD1 mutations sensitize MSS tumors to ICIs, challenging the dominance of microsatellite instability (MSI) as an immunotherapy predictor. Integrating structural insights, molecular mechanisms, and clinical data, this review positions POLD1 mutations as both a driver of cancer progression and a promising biomarker, redefining therapeutic possibilities in precision oncology.

## 1 Introduction

DNA polymerase delta (Pol δ) is a multi-subunit enzyme complex consisting of four subunits: POLD1, POLD2, POLD3, and POLD4. It plays a critical role in DNA replication, specifically in synthesizing the lagging strand, and is also involved in multiple DNA repair pathways, including base excision repair, double-strand break repair, and mismatch repair (MMR) (1, 2). The catalytic subunit of Pol δ, POLD1, ensures replication fidelity through its 3’–5’ exonuclease domain, which proofreads newly synthesized DNA with an error rate of approximately 10^-6^ mutations per base (3). This proofreading function is essential for maintaining genomic stability, as it prevents the accumulation of mutations that could disrupt cellular homeostasis.

However, when this proofreading function is compromised by mutations in POLD1, particularly in its exonuclease domain, Pol δ contributes to genomic instability—a hallmark of cancer. This genomic instability drives tumorigenesis by fueling the persistent accumulation of mutations. The impact of POLD1 mutations in cancer is complex, with distinct roles in germline and somatic contexts. Germline mutations in the exonuclease domain, such as p.S478N, underlie polymerase proofreading-associated polyposis (PPAP), a hereditary syndrome marked by early-onset colorectal cancer and other malignancies due to defective proofreading and elevated mutation rates (3, 4). Somatic mutations, exemplified by p.L474P, further amplify this mutator phenotype, generating ultra-hypermutated tumors with tumor mutation burden (TMB) often exceeding 100 mutations per megabase (5).

Surprisingly, while microsatellite-stable (MSS) tumors are generally resistant to immune checkpoint inhibitors (ICIs) due to low immunogenicity, POLD1-mutated MSS tumors defy this trend. These tumors exhibit robust immunogenicity, driven by high TMB and neoantigen loads, resulting in enhanced T-cell infiltration and ICI responsiveness (6). This paradox challenges the conventional reliance on microsatellite instability (MSI) as a prerequisite for ICI efficacy and positions POLD1 mutations as a novel bridge between genomic instability and immune recognition.

This review synthesizes recent advances in understanding how POLD1 mutations orchestrate both tumorigenesis and immunotherapy responses, proposing a central hypothesis: POLD1 mutations redefine the therapeutic landscape of MSS tumors by reshaping their mutational landscape and immune microenvironment, independent of traditional MSI pathways. We first analyze the molecular mechanisms by which domain-specific POLD1 mutations—whether in the exonuclease, polymerase, or regulatory regions—drive genomic instability and fuel cancer progression across diverse clinical contexts, from PPAP to sporadic malignancies. We then explore how these mutations, by generating distinct mutational signatures (e.g., SBS10d) and high TMB, enhance tumor immunogenicity, rendering MSS tumors vulnerable to ICIs despite their stable microsatellites. Finally, we integrate structural biology, tumor evolution, and clinical oncology insights to argue that POLD1’s dual role as a mutator and immunogenic trigger offers a new paradigm for precision cancer therapy. By elucidating POLD1’s multifaceted contributions—from replication stress to immune evasion—we aim to illuminate its potential as a predictive biomarker and therapeutic target, paving the way for tailored strategies that exploit its vulnerabilities in the fight against cancer.

## 2 POLD1 biology: structure and function

### 2.1 Structural determinants of POLD1 function

**Figure 1:**
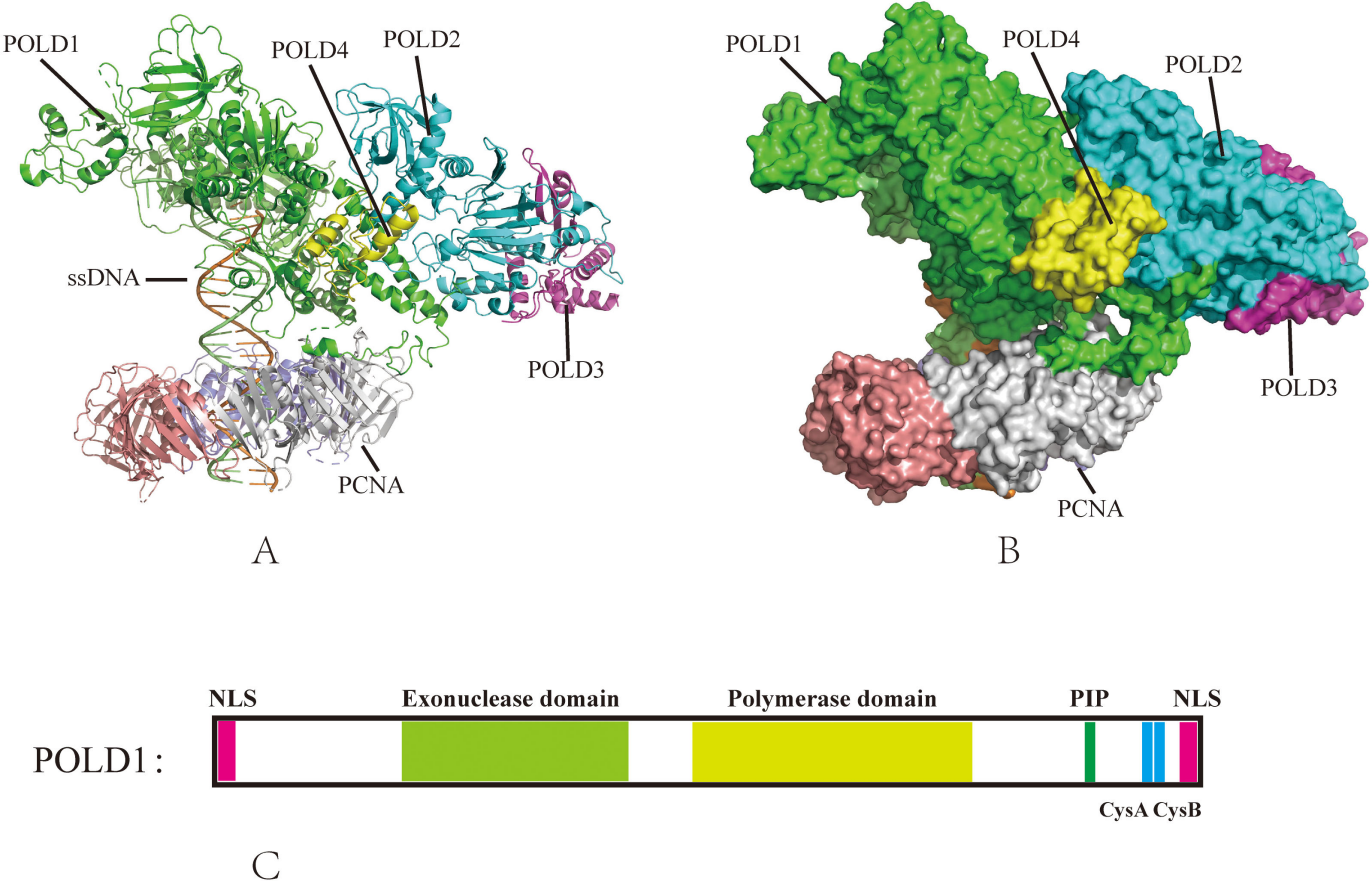
The structure of Pol δ–DNA–PCNA complex. **(A)** The published Pol δ–DNA-PCNA secondary structure (PDB ID: 6TNY); **(B)** The published Pol δ–DNA–PCNA three-dimensional structure (PDB ID: 6TNY); **(C)** Domain organization of POLD1, nuclear localization sequence (NLS), PCNA-interacting protein (PIP), cysteine-rich metal-binding sites CysA and CysB.

POLD1, the catalytic subunit of DNA polymerase delta (Pol δ), is a multi-domain protein (125 kDa) critical for DNA synthesis and proofreading (7). It comprises three key domains: the N-terminal exonuclease domain, the central polymerase domain, and the C-terminal domain with a zinc finger (CysA), a [4Fe-4S] cluster (CysB), and a non-canonical Proliferating Cell Nuclear Antigen (PCNA)-interacting (PIP) box (2). Each domain contributes uniquely to replication fidelity and repair, with distinct structural and functional properties (**Figure 1**).

#### 2.1.1 N-terminal exonuclease domain (residues 304–533): guardian of replication fidelity

The exonuclease domain executes 3′-5′ hydrolytic excision of mismatched nucleotides during replication (3, 8). Structural analyses reveal that conserved acidic residues Asp316 and Glu318 in the ExoI motif coordinate magnesium ions essential for catalytic activity, while Asp402 (ExoII) and Asp515 (ExoIII) stabilize substrate positioning (9, 10). The DEDD motif (Asp316-Glu318-Asp402-Asp515) represents a mutational hotspot, particularly in mismatch repair-deficient backgrounds. For instance, the p.D316H variant eliminates exonuclease activity and is linked to endometrial carcinoma with ultramutated genomes (11).

#### 2.1.2 Central polymerase domain (residues 579–974): the synthetic engine

This domain houses the DNA polymerase active site, where conserved residues Asp602 (Motif A) and Asp757 (Motif C) coordinate dNTP incorporation during strand elongation [12]. Specific substitutions, including the p.V759I variant tentatively associated with Ashkenazi Jewish founder effects, induce domain-wide conformational shifts that alter polymerase fidelity (13). Disruption of PCNA-binding residues, such as p.K931A in the thumb subdomain, reduces template-primer contacts and processivity by >80% (9). Such defects mirror observations in *S. cerevisiae*, where analogous mutations impair replication fork progression due to defective PCNA-polymerase cooperation (14).

#### 2.1.3 C-terminal regulatory region (residues 901–1107): integration of stability and processivity

The C-terminal regulatory region coordinates replication mechanics through conserved structural elements: the CysA zinc finger (residues 901–1000), which maintains holoenzyme stability via Zn²^+^-dependent subunit assembly; the CysB motif, where a [4Fe-4S] cluster mediates oxidative stress adaptation and DNA binding – structural disruptions (e.g., p.I1070N) in this region precipitate misfolding syndromes marked by accelerated progeroid phenotypes and immune dysfunction (15); and finally, Non-canonical PIP box (residues 1001–1005), which mediates binding to PCNA through a unique two-fork plug (Leu1002-Phe1005). Deletions in the PIP box (e.g., p.L1002A) disrupt PCNA tethering, reducing processivity by >90% in heparin-trapping assays (16). The C-terminal regulatory region plays a crucial role in coordinating stability and processivity during replication, particularly through its CysA zinc finger motif, which facilitates subunit assembly, and the CysB motif, which helps POLD1 adapt to oxidative stress—an essential feature for cancer cells dealing with high mutagenic pressure.

### 2.2 Regulatory networks and expression dysregulation in cancer

The expression and activity of POLD1 are governed by a complex interplay of transcriptional, post-transcriptional, and post-translational mechanisms, which collectively shape its paradoxical roles in maintaining genomic stability and driving oncogenic processes. Transcriptionally, E2F1 and Sp1/Sp3 activate POLD1 during S phase via GC-rich promoter elements, while genotoxic stress triggers p53/p21-mediated repression through Sp1/DNMT1 downregulation and DREAM complex disruption (17–19). CTCF maintains promoter accessibility, linking age-related POLD1 decline to senescence (20).

POLD1 is highly expressed in various cancers, including bladder, breast, endometrial, colorectal, lung, and hepatocellular carcinoma, as evidenced by both clinical studies and TCGA database analyses (16, 21–25) However, its expression patterns yield paradoxical clinical outcomes depending on tumor context. In hepatocellular carcinoma (HCC), TP53 mutations drive POLD1 overexpression, which correlates with advanced tumor stage, vascular invasion, and poor prognosis (22). This aligns with its role in promoting replication under oncogenic stress, where high POLD1 levels exacerbate genomic instability in rapidly proliferating tumors. Strikingly, in clear cell renal cell carcinoma (ccRCC), elevated POLD1 expression paradoxically associates with favorable survival outcomes, potentially reflecting its function in maintaining replication fidelity in slower-growing malignancies with lower baseline replication stress (26). These divergent phenotypes underscore the dual nature of POLD1 in cancer biology: while its overexpression may drive replication-associated mutagenesis in aggressive tumors, it can paradoxically act as a stabilizing force in malignancies where replication demands are less acute.

## 3 Pathological implications of POLD1 mutations

### 3.1 Mutation topography dictates clinical phenotypes

The spatial distribution of POLD1 mutations across different functional domains determines their distinct pathological consequences through domain-specific mechanisms that affect genomic stability and cellular homeostasis. POLD1 maintained the genomic fidelity through its 3’–5’ exonuclease proofreading activity, making domain-specific mutations particularly consequential for cellular function.

The exonuclease domain mutations (e.g., p.L474P, p.L472P, P286R, S459F) represent the most clinically significant alterations, disrupting proofreading function and leading to catastrophic accumulation of somatic mutations that often exceed 100 mutations per megabase (mut/Mb), a hallmark of ultra-hypermutation (16, 27–31). This genomic instability paradoxically transforms tumors into immunogenic hotspots, creating a unique interplay between mutagenesis and immune recognition. Structural studies reveal that exonuclease domain mutations such as P286R and S459F distort the DNA-binding groove, destabilizing primer-template alignment and exacerbating replication errors (32).

In contrast, polymerase-domain variants (e.g., p.L606M) operate through distinct mechanisms, hijacking replication stress signaling pathways rather than directly compromising proofreading fidelity, potentially favoring tumorigenesis in glioma and polyposis-prone tissues (33). A third category, germline truncating mutations (p.S605del) that spare the exonuclease domain, escape canonical tumorigenesis but provoke MDPL syndrome (mandibular hypoplasia, progeroid lipodystrophy) via impaired PCNA interaction and systemic replication catastrophe (8). POLD1 exonuclease domain mutations induce distinct mutational signatures dominated by single-nucleotide variants (SNVs), particularly TCT>TAT (COSMIC signature SBS10a) and TCG>TTG (SBS10b) transitions, differing from frameshift-driven patterns typical of MSI (34, 35). These mutations are often clonal and early events, preceding other driver mutations, which may explain the widespread genomic heterogeneity observed in these tumors (36).

These mutations result in TMB levels surpassing those of mismatch repair-deficient (dMMR)/MSI-high (MSI-H) cancers, with median TMB values exceeding 150 mut/Mb in colorectal and endometrial cancers (31, 32). This hypermutated phenotype predominantly affects colorectal and endometrial malignancies, generating SBS10d mutational signatures arising from somatic LOH (loss of heterozygosity, loss of the wild-type allele) (16, 27, 28). Although POLD1 mutations are rare in TCGA(~1–2%) (34), they present a unique paradox: they drive genomic instability while paradoxically enhancing immunogenicity, particularly in MSS tumors traditionally resistant to ICIs. Notably, POLD1-mutant tumors are predominantly MSS across most cancer types, yet exhibit significant tissue-specific variation. For instance, in stomach adenocarcinoma (STAD), >90% show co-occurring MSI-H (see Section 5), while in colorectal and endometrial cancers, >98% maintain MSS status despite ultra-hypermutation (29, 37).

Despite MSS status, POLD1-mutant tumors display robust immune infiltration, including elevated CD8+ T cells and PD-L1 expression (35). The sheer volume of clonal neoantigens generated by ultra-hypermutation likely drives this immunogenicity, bypassing the reliance on MSI-induced frameshift neoantigens (28, 38). For example, POLD1-mutant MSS colorectal cancers exhibit higher tumor-infiltrating lymphocyte density and cytotoxic T-cell markers compared to POLD1 wild-type MSS tumors (16, 34). This functional dichotomy underscores domain-selective vulnerabilities within POLD1: exonuclease perturbations ignite immunogenic mutagenesis through hypermutation, whereas polymerase and C-terminal domain defects disrupt replication homeostasis in developmentally sensitive lineages. The genomic instability paradoxically transforms tumors into immunogenic hotspots, creating a unique interplay between mutagenesis and immune recognition that has significant therapeutic implications.

### 3.2 Germline vs. somatic mutations

Germline mutations in POLD1 are present from embryogenesis, leading to systemic effects across multiple tissues throughout development. Variants like p.Ser478Asn and p.Leu474Pro impair Pol δ’s proofreading capacity, increasing base substitution and insertion-deletion mutation rates (39, 40). This continuous accumulation, detectable in normal crypts, endometrial glands, and even sperm, reflects a systemic replication defect originating in the embryo. Variants like S478N, inherited dominantly, elevate mutation rates across all tissues lifelong—152 SBS per year versus 49 in healthy controls—contrasting with the sporadic, tumor-specific onset of somatic mutations (41). This results in an ultramutated phenotype characterized by COSMIC Signature 10 variants, consistent with somatic POLD1-mutant profiles (42).

Molecularly, the apparent contradiction between functional studies suggesting near-haplosufficiency and the dominant inheritance pattern of POLD1-related cancer predisposition (like PPAP) warrants clarification. Functional data indicate that heterozygous exonuclease domain mutations alone cause only a subtle increase in mutation rate (<15%) in somatic cells, implying near-haplosufficiency under normal conditions, potentially due to extrinsic proofreading by wild-type Pol δ complexes (27, 43). However, the dominant cancer risk associated with germline heterozygous mutations strongly suggests that a single mutant allele is sufficient to significantly increase cancer susceptibility. This paradox is resolved by the frequent occurrence of somatic LOH at the POLD1 locus in tumor tissue. LOH eliminates the wild-type allele, creating a functionally homozygous or hemizygous state for the mutant allele, which catastrophically compromises proofreading. This two-hit mechanism (germline mutation + somatic LOH) explains both the dominant inheritance pattern and the tissue-specific vulnerability observed in PPAP, where rapid cell turnover increases the likelihood of acquiring the somatic LOH event (27, 29, 37, 40).

In contrast, somatic POLD1 mutations, mostly in the exonuclease domain, emerge in adulthood, driving tumorigenesis via proofreading deficits. Studies identify variants like p.S478N in MSS colorectal tumors, recapitulate the ultra-hypermutation and SBS signatures observed in germline contexts, but differ from the insertion-deletion patterns of Lynch syndrome (44, 45). Functional assays in yeast, using the Pol3-C462N strain (corresponding to human p.S478N), reveal a 12-fold increase in mutation rate, confirming that proofreading loss amplifies somatic SBS accumulation (44). This localized effect contrasts sharply with the systemic impact of germline mutations.

The molecular impact of these mutations is highly context-dependent, often synergizing with mismatch repair (MMR) deficiency to produce complex mutational profiles. For instance, in MSS tumors, somatic POLD1 exonuclease domain mutations alone generate hypermutation signatures like SBS10, characterized by C:G→A:T and C:G→T:A transitions (44). However, when combined with MMR defects, they yield distinct signatures such as SBS20, reflecting a compounded effect on genomic stability. A notable example involves a serrated polyposis syndrome (SPS) patient harboring a germline POLD1 frameshift (p.Lys648fs*46) outside the exonuclease domain, where somatic MMR loss led to ultra-hypermutation (TMB > 117 mut/Mb). Despite loss of heterozygosity of the wild-type allele in the tumor, suggesting a haploinsufficient state, this case lacked POLD1-specific signatures (e.g., SBS10c/d), indicating MMR dominance over POLD1’s contribution (40). This demonstrates the hierarchical nature of DNA repair pathway interactions and their relative contributions to tumor mutagenesis.

### 3.3 Domain-specific disease phenotypes (PPAP, MDPL)

The functional compartmentalization of POLD1 creates distinct pathogenic mechanisms, where mutations in different domains produce entirely different clinical syndromes, providing compelling evidence for domain-specific functional requirements.

#### 3.3.1 Polymerase domain mutations and replication stress in MDPL

Germline mutations in the polymerase domain of POLD1 are the molecular basis of MDPL, a rare autosomal dominant syndrome (OMIM #615381). The most prevalent mutation, p.Ser605del, accounts for approximately 77–85% of cases, as reported across studies, and resides in the conserved motif A of the polymerase active site (39, 46). Functional studies reveal that this in-frame deletion abolishes polymerase activity while retaining partial exonuclease function, disrupting DNA synthesis during replication (39). The molecular consequences of this mutation are well-characterized through functional assays. *In vitro* assays using Escherichia coli expressing p.Ser605del demonstrate that the mutant Pol δ binds DNA but fails to catalyze nucleotide incorporation, leading to stalled replication forks (47). This replication stress triggers double-strand breaks (DSBs), as evidenced by increased γH2AX foci in MDPL patient-derived fibroblasts, persisting even after DNA damage induction with cisplatin or X-irradiation (39).

Beyond the predominant p.Ser605del mutation, rare polymerase domain variants illustrate the structure-function relationship. For instance, p.Ile1070Asn in the CysB motif, intensify this phenotype by disrupting iron-sulfur cluster binding, critical for holoenzyme stability. Structural modeling predicts that p.Ile1070Asn alters the CysB loop topology, weakening interactions with accessory subunits (e.g., POLD2, POLD4), while milder variants like p.Ser1073Arg have subtler electrostatic effects, correlating with less severe clinical manifestations (40). These findings underscore how polymerase domain mutations drive MDPL through replication stress and structural disruption.

#### 3.3.2 PPAP: exonuclease domain deficiency

In contrast, germline mutations in the exonuclease domain of POLD1 lead to PPAP, an autosomal dominant cancer predisposition syndrome with fundamentally different clinical manifestations (48). PPAP is characterized by early-onset colorectal adenomas and carcinomas, with studies documenting variable but significant cancer risks in affected families (49). The syndrome also features increased risks for endometrial, ovarian, and brain tumors, reflecting the systemic impact of defective DNA proofreading (50).

Molecularly, PPAP-associated mutations disrupt the exonuclease active site, leading to loss of proofreading function, with specific variants like p.Leu474Pro and p.Ser478Asn reducing proofreading efficiency by 10–100 fold in functional assays (29). This defect causes the accumulation of mutant genes in cells, leading to accelerated mutation accumulation, particularly in rapidly dividing tissues like colonic epithelium, where the mutation burden can exceed 100 mutations/Mb (27). Unlike MDPL, which manifests with developmental and metabolic abnormalities, PPAP primarily increases cancer susceptibility without significant developmental phenotypes, highlighting the distinct consequences of domain-specific mutations.

#### 3.3.3 Mechanistic divergence and clinical heterogeneity

The domain-specific effects of POLD1 mutations underpin their clinical diversity. Polymerase domain defects (MDPL) induce replication stress, DSBs, driving early-onset progeroid features (39, 46). Exonuclease domain mutations (PPAP, somatic cancers) compromise proofreading, elevating mutation rates in proliferative tissues (27, 39, 40). In somatic MSS cancers, POLD1 exonuclease domain mutations uniquely mimic MSI-H hypermutation, boosting neoantigen load and distinguishing them from typical MSS tumors (6, 51). Somatic mutations’ dependence on secondary hits (e.g., MMR loss) delays onset, contrasting with germline mutations’ earlier manifestations. This mechanistic divergence—replication failure versus proofreading loss—illustrates how POLD1 mutations bridge molecular dysfunction to heterogeneous phenotypes, from childhood MDPL to adult-onset cancers.

Recent studies have proposed the term “POLE/POLD1-associated tumor syndrome” to encompass a broader clinical spectrum beyond classical PPAP, recognizing that some patients carrying germline mutations in POLE or POLD1 may develop tumors without evidence of polyposis (33, 52). This expanded classification reflects the growing appreciation for the heterogeneous phenotypes associated with polymerase proofreading deficiencies and underscores the need for more inclusive diagnostic criteria.

## 4 POLD1-driven genomic instability

### 4.1 Molecular mechanisms of POLD1-mediated genomic instability

POLD1 mutations drive genomic instability through distinct molecular mechanisms that extend beyond simple proofreading defects. Recent studies have revealed that POLD1 deficiency disrupts the spatial-temporal coordination of replication factories, leading to aberrant origin firing and replication timing (17). Tumini et al. demonstrated that POLD1 depletion significantly reduces the density of active replication origins, forcing cells to rely on fewer origins that must travel longer distances, thereby increasing the probability of replication fork collapse (53). This origin paucity creates regions of under-replicated DNA that persist into mitosis, forming ultrafine anaphase bridges and promoting chromosomal rearrangements. Importantly, POLD1 mutations exhibit allele-specific effects on genomic stability; while exonuclease domain mutations drive point mutation accumulation via misincorporation, polymerase domain variants induce replication fork stalling and double-strand breaks through defective Okazaki fragment maturation (8).

### 4.2 Synergistic interactions with DNA repair pathways

POLD1-driven genomic instability does not operate in isolation but rather interacts synergistically with other DNA repair mechanisms, particularly the MMR pathway. While POLD1 mutations and MMR deficiency can independently induce genomic instability, their co-occurrence creates a synergistic effect that dramatically accelerates mutagenesis (29). This interaction follows a specific evolutionary trajectory: initial POLD1 proofreading deficiency generates a moderate increase in mutation rate, which subsequently leads to the acquisition of secondary mutations in MMR genes, further amplifying genomic instability in a feed-forward loop (35). Specific POLD1 mutations, such as somatic p.S478N and germline p.L474P, impair Pol δ proofreading, leading to ultramutated tumors with tumor mutation frequent loads exceeding 100 variants/Mb and characteristic SBS10d signatures (54, 55). Notably, these mutations frequently co-occur with secondary somatic inactivation of MMR genes, as observed in ultramutated Lynch Syndrome patients, amplifying mutation loads through a synergistic feed-forward mechanism (54). This interaction induces MSI signatures, despite the predominantly MSS phenotype of POLD1-mutated tumors, highlighting a unique evolutionary trajectory where Pol δ infidelity triggers secondary MMR defects (56).

Intriguingly, despite this interaction, most POLD1-mutated tumors maintain MSS, distinguishing them from classical MMR-deficient cancers characterized by microsatellite instability (MSI) (6). This paradoxical observation suggests that POLD1-driven genomic instability follows a distinct evolutionary path, preferentially accumulating single-nucleotide variants rather than the insertion-deletion mutations typical of MMR deficiency. The maintenance of microsatellite stability despite high mutational burden represents a unique feature of POLD1-mutated cancers and has significant implications for their biological behavior and therapeutic responsiveness.

Beyond MMR, POLD1 mutations also impact other DNA repair pathways, including base excision repair and double-strand break repair, where POLD1 normally plays crucial roles in gap-filling and strand displacement synthesis (32). The compromised function of these repair pathways further exacerbates genomic instability, creating a state of “repair crisis” that fuels ongoing mutagenesis and chromosomal aberrations.

### 4.3 Replication stress and fork dynamics

**Figure 2:**
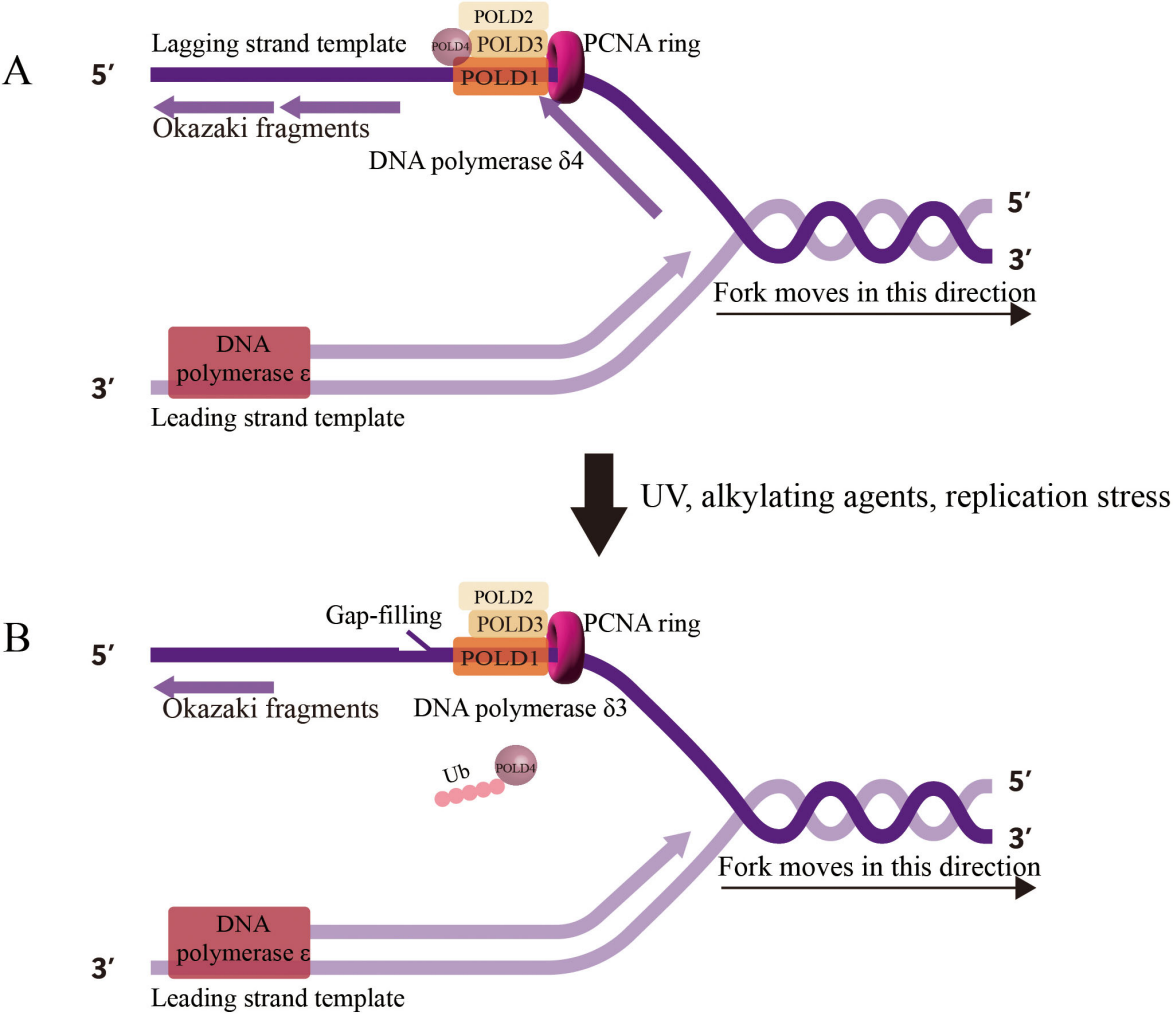
Human DNA Polymerase δ Holoenzyme on the Genome. **(A)** Pol δ4 (POLD1, POLD2, POLD3, POLD4) synthesizes Okazaki fragments on the lagging strand during normal replication, **(B)** Under replication stress, Pol δ3 (lacking POLD4, degraded via ubiquitination) performs gap-filling and lagging strand synthesis, reflecting its role in error-prone repair.

POLD1 mutations fundamentally alter replication fork dynamics, inducing chronic replication stress that fuels genomic instability. Under normal conditions, POLD1 coordinates with POLD3 to maintain replication fork stability and processivity (53). However, when POLD1 function is compromised, replication forks exhibit increased stalling, reversed fork structures, and ssDNA accumulation (57). POLD1 transitions between different functional states—from the complete Pol δ4 complex (POLD1, POLD2, POLD3, and POLD4) to the Pol δ3 form (lacking POLD4)—in response to replication challenges (**Figure 2**) (58, 59). This transition, mediated by the ubiquitin-proteasome pathway and regulated by the ATR-CHK1 signaling axis, represents an adaptive mechanism to navigate replication obstacles (12).

However, POLD1 mutations disrupt this delicate balance, leading to inappropriate activation of error-prone repair mechanisms such as break-induced replication (BIR) (12). BIR, while rescuing stalled replication forks, introduces extensive genomic alterations, including long-tract gene conversions and loss of heterozygosity (LOH). Indeed, LOH at the POLD1 locus is frequently observed in tumors with heterozygous POLD1 mutations, resulting in the complete loss of proofreading capacity and accelerated genomic instability (37).

The replication stress induced by POLD1 mutations also manifests as increased double-strand breaks (DSBs), particularly at common fragile sites and regions with complex secondary structures (36, 60). These DSBs, when repaired through error-prone mechanisms like alternative end-joining, generate chromosomal rearrangements that further destabilize the genome. The accumulation of such structural variations represents a distinct dimension of POLD1-driven genomic instability, complementing the hypermutation phenotype and contributing to the overall genomic instability characteristic of POLD1-mutated cancers.

## 5 Immune evasion mechanisms in POLD1-mutant tumors

The interplay between POLD1 mutations and immune evasion represents a critical aspect of tumor biology with significant implications for therapeutic strategies. While POLD1 mutations are associated with increased TMB and potential neoantigen formation that could enhance immune recognition, paradoxically, these tumors often develop sophisticated mechanisms to evade immune surveillance.

A primary mechanism of immune evasion in POLD1-mutant tumors is the upregulation of immune checkpoint molecules, notably programmed death-ligand 1 (PD-L1). PD-L1 expression on tumor cells interacts with PD-1 on T cells, suppressing T cell activity and dampening the antitumor immune response. In stomach adenocarcinoma (STAD), POLD1-mutant tumors exhibit significantly higher PD-L1 mRNA expression compared to wild-type tumors (P=0.0072), indicative of adaptive immune resistance (61). This mechanism allows tumors to counteract the immune activation triggered by their high neoantigen load. Similarly, in colorectal cancer, POLD1-mutant tumors demonstrate enhanced responsiveness to ICIs, with an overall response rate of 89% compared to 54% in dMMR/MSI-H tumors (P=0.01), suggesting that PD-L1-mediated immune suppression is a critical evasion strategy that can be targeted therapeutically (31).

POLD1-mutant tumors may also foster an immune-excluded tumor microenvironment (TME), characterized by the presence of immune cells in the tumor stroma but limited infiltration into the tumor bed. This spatial barrier prevents effective interaction between immune effector cells and tumor cells, thereby facilitating immune evasion. Analysis using databases like TIMER has revealed that POLD1 expression, and by extension mutations, correlates with increased infiltration of immunosuppressive cells, such as regulatory T cells (Tregs) and myeloid-derived suppressor cells (MDSCs), in cancers like ccRCC (24). These cells contribute to an immunosuppressive TME by secreting inhibitory cytokines (e.g., TGFβ, IL-10) and downregulating T cell function, further limiting immune penetration. In STAD, POLD1-mutant tumors exhibit a TME immune type I profile, with high PD-L1 and CD8A expression (45.16% vs. 33.43% in wild-type, P=0.004), suggesting a TME that is both inflamed and resistant due to adaptive immune mechanisms (61).

The interplay between POLD1 mutations and DNA repair deficiencies, such as MMR defects, further complicates immune evasion. In STAD, 90% of POLD1-mutant tumors are MSI-H, with a higher mutation rate in MMR-related genes (e.g., MLH1, MLH3) compared to wild-type tumors (P<0.001) (61). This synergy can amplify TMB, enhancing neoantigen production, but also contributes to immune evasion by promoting a complex mutational landscape that may include mutations in genes associated with immune resistance, such as PTEN or TP53 (30, 62). For instance, PTEN mutations, frequently observed in POLD1-mutant mouse models, disrupt genome stability and immune signaling, potentially enhancing immune evasion by impairing antigen presentation or T cell priming (62).

Publicly available databases like TIMER and TISIDB provide critical insights into the immune landscape of POLD1-mutant tumors. These tools estimate immune cell infiltration based on gene expression data, revealing correlations between POLD1 alterations and immune cell types. For example, in ccRCC, high POLD1 expression is associated with increased infiltration of Tregs, MDSCs, and CD56bright NK cells, which have weaker cytotoxicity compared to CD56dim NK cells, contributing to an immunosuppressive TME (24). TIMER analyses also show significant correlations between POLD1 expression and immune checkpoint molecules (e.g., PD-1, CTLA-4, LAG3) and chemokines (e.g., CCL5, CXCL13), suggesting that POLD1 mutations may modulate the TME to favor immune evasion (24). These findings underscore the utility of such databases in elucidating the immune dynamics of POLD1-mutant tumors and guiding therapeutic strategies.

## 6 Therapeutic implications of POLD1 mutations in cancer

### 6.1 POLD1 as a predictive biomarker for immunotherapy

**Figure 3:**
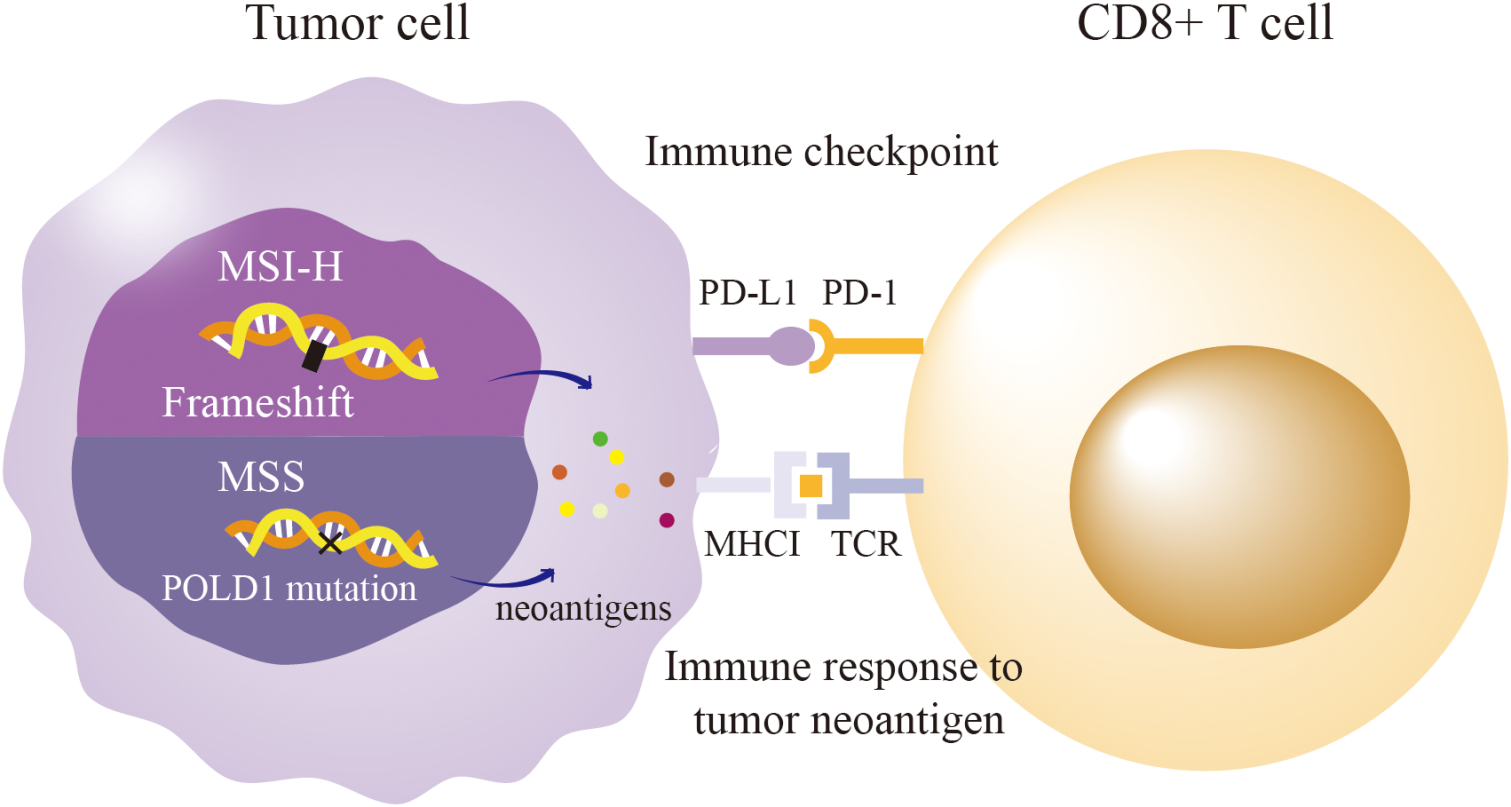
POLD1 Mutation Paradox: Immunogenicity in MSS vs. MSI-H Tumors. MSI-H: Mismatch repair defects lead to frameshift mutation, producing neoantigens and driving T-cell infiltration and PD-L1/PD-1 signaling. MSS: POLD1 exonuclease mutations In review cause high tumor mutation burden via single-nucleotide variants, generating neoantigen that trigger CD8+ T-cell responses and PD-L1/PD-1 checkpoint activation despite MSS status.

ICI efficacy typically correlates with TMB and MMR deficiency/MSI-H status, yet POLD1 mutations enable MSS tumors to respond by inducing a hypermutated phenotype (**Figure 3**) (28, 38). Pan-cancer analyses reveal that patients with POLD1-mutated tumors achieve superior outcomes with ICIs, including prolonged overall survival (34 months for POLD1-mutated vs. 18 months for wild-type MSS tumors) and response rates > 80% in some studies (31, 63). For instance, in colorectal cancer, POLD1 exonuclease domain mutations (e.g., p.S478N, p.L474P) correlate with elevated TMB and robust CD8+ T-cell infiltration, driving durable responses to anti-PD-1 therapy despite MSS status (64). Similarly, in endometrial cancer, the p.D316H variant in the DEDD motif is associated with a T-cell-inflamed gene expression profile, predicting pembrolizumab sensitivity with a complete response in a documented case (11). Remarkably, polymerase domain mutations (e.g., p.L606M) also correlate with ICI efficacy, suggesting that POLD1’s influence transcends proofreading defects (33). The Acsé Nivolumab trial reinforces this, showing mutations in DNA-binding or catalytic regions yield responses rivaling those in traditionally responsive tumors (58.7% vs. 23.8% in wild-type) (65). These findings underscore POLD1’s potential as an independent biomarker for ICI response, broadening the therapeutic horizon beyond MSI-H paradigms.

### 6.2 Synergistic effects with other genetic alterations

The therapeutic implications of POLD1 mutations are amplified by their interplay with co-occurring genetic alterations. Notably, co-mutations with PBRM1, a tumor suppressor, enhance ICI efficacy in POLD1-mutated tumors. In advanced solid tumors, POLD1/PBRM1 co-mutations yielded higher response rates (70% vs. 40% for POLD1 alone), driven by elevated TMB and a T-cell-permissive microenvironment (66). PBRM1 loss sensitizes tumor cells to interferon-γ-mediated killing, complementing the neoantigen-driven immunogenicity of POLD1 mutations (67). This synergy suggests that POLD1/PBRM1 co-mutation could serve as a composite biomarker, identifying a subset of MSS patients likely to benefit from immunotherapy.

POLD1 also frequently co-occurs with oncogenic drivers like APC, BRCA2, and MYC, notably in colorectal and bladder cancers (16, 36). In bladder cancer, POLD1 stabilizes MYC by inhibiting its degradation via FBXW7, promoting proliferation and metastasis (16). While this pro-tumorigenic axis complicates prognosis, it also hints at combinatorial strategies: targeting MYC alongside ICIs could exploit POLD1-driven immunogenicity while curbing tumor growth. These interactions highlight the need for comprehensive genomic profiling to tailor therapies based on POLD1’s mutational context.

### 6.3 Clinical trials targeting POLD1/POLE mutations

Several clinical trials are investigating the therapeutic potential of POLD1 and POLE mutations, particularly their role in predicting ICI response in hypermutated tumors. **Table 1** summarizes key ongoing or recently completed trials focused on POLD1/POLE mutations and their impact on immunotherapy outcomes.

**Table 1:** Clinical trials investigating POLD1/POLE mutations and immunotherapy response.

Trial ID	Title/Focus	Phase	Status	Intervention	Target Population
NCT03810339	Pembrolizumab in POLE/POLD1 Mutated Advanced Solid Tumors	II	Recruiting	Pembrolizumab (Anti-PD-1)	Advanced solid tumors with pathogenic POLE/POLD1 mutations
NCT05103969	Immunotherapy in POLE/POLD1 Mutated Colorectal Cancer	II	Active, not recruiting	Nivolumab + Ipilimumab	Microsatellite stable metastatic colorectal cancer with POLE/POLD1 mutations Solid tumors with ultra-high mutation burden (≥100 mutations/Mb)
NCT03461952	Atezolizumab in Ultra-Hypermutated Tumors	II	Active, not recruiting	Atezolizumab (Anti-PD-L1)	Solid tumors with ultra-high mutation burden (≥100 mutations/Mb)
NCT04969029	Combination Immunotherapy in POLE/POLD1 Cancers	I/II	Recruiting	Dostarlimab + Niraparib	Advanced solid tumors with POLE/POLD1 mutations
NCT03012581	Nivolumab in Hypermutated Solid Tumors	II	Completed	Nivolumab (Anti-PD-1)	Solid tumors with high tumor mutation burden
NCT02693535	TAPUR Study: Targeted Therapy Based on Tumor Genetics	II	Active, not recruiting	Multiple targeted agents including ICIs	Advanced cancers with actionable genomic variants

### 6.4 Controversies and context-dependent prognosis

Despite its promise, the prognostic and therapeutic significance of POLD1 remains contentious, varying across cancer types. In HCC, POLD1 overexpression, often driven by TP53 mutations, correlates with advanced staging, vascular invasion, and poor survival, suggesting a tumor-promoting role resistant to conventional therapies (22). Similarly, in triple-negative breast cancer and endometrial carcinoma, high POLD1 expression is linked to aggressive phenotypes and reduced survival, potentially reflecting its role in sustaining replication under oncogenic stress (68). Conversely, in ccRCC elevated POLD1 expression paradoxically associates with favorable survival, possibly due to preserved replication fidelity in less aggressive tumors (26). This dichotomy underscores a critical challenge: POLD1’s therapeutic relevance is highly context-dependent, influenced by tumor biology, mutation type, and coexisting alterations.

The predictive value of POLD1 mutations for ICI efficacy also faces scrutiny. While high TMB in POLD1-mutated tumors enhances immunogenicity, the lack of MSI-H signatures in most cases raises questions about the mechanisms driving immune recognition. Some studies argue that clonal neoantigens, rather than frameshift mutations typical of MSI-H, underlie this effect, yet the precise neoantigen profiles remain poorly characterized (69). Moreover, the rarity of POLD1 mutations (1-2% in TCGA cohorts) limits large-scale validation, necessitating prospective trials to confirm its utility as a standalone biomarker.

### 6.5 Emerging therapeutic strategies

POLD1’s biology inspires innovative approaches. Its mutational signatures (e.g., SBS10a, SBS10b) suggest neoantigen vaccines targeting specific base transitions, potentially boosting T-cell responses in MSS tumors (34, 35). Second, POLD1’s role in replication stress opens avenues for synthetic lethality. Mutations like p.S605del in the polymerase domain, which impair DNA synthesis and induce double-strand breaks (DSBs), render cells reliant on ATR-CHK1 signaling for fork stabilization (70). Inhibitors of ATR or CHK1, such as berzosertib or prexasertib, could selectively kill POLD1-mutant tumor cells by exacerbating replication collapse, a strategy already showing promise in POLE-mutated cancers (71). Combining these agents with ICIs might further enhance efficacy by increasing tumor cell death and neoantigen release. Finally, targeting POLD1’s regulatory network offers indirect therapeutic leverage. In bladder cancer, POLD1’s stabilization of MYC suggests that MYC inhibitors (e.g., KJ-Pyr-9) could disrupt this axis, while miR-155 agonists might suppress POLD1 expression by downregulating FOXO3a, reducing replication fidelity in tumor cells (16, 72). These approaches, though speculative, illustrate how POLD1’s molecular dependencies could be exploited to design combination therapies.

## 7 Future perspectives

To fully harness POLD1’s therapeutic potential, prospective clinical trials are critical to validate its role as an immunotherapy biomarker, particularly in MSS tumors with high TMB. Biomarker-driven trials, such as those stratifying patients by POLD1 mutation domain (exonuclease vs. polymerase) and co-mutational status (e.g., PBRM1, APC, or BRCA2), could refine predictive models and optimize patient selection for ICIs. For instance, basket trials targeting POLD1/PBRM1 co-mutations could identify MSS patients likely to benefit from anti-PD-1 therapies, leveraging the synergistic immunogenicity of these alterations. Additionally, combinatorial strategies hold promise: combining ICIs with ATR/CHK1 inhibitors like berzosertib could exploit POLD1-driven replication stress, enhancing tumor cell death and neoantigen release. Neoantigen vaccines tailored to POLD1-specific mutational signatures (e.g., SBS10a/b) could further boost T-cell responses in MSS tumors. Integrating POLD1 profiling into routine next-generation sequencing panels would enhance its clinical accessibility, given its low prevalence (1–2% in TCGA cohorts). Long-term, these approaches could redefine treatment paradigms for refractory MSS cancers, offering hope for improved outcomes in cancers historically resistant to immunotherapy.

## 8 Conclusion

POLD1 mutations emerge as dynamic architects of cancer biology, intertwining genomic instability with immune evasion while unveiling novel therapeutic avenues. This review demonstrates how these mutations—whether in the exonuclease, polymerase, or regulatory domains—fuel diverse clinical outcomes, from hereditary conditions like PPAP to sporadic ultra-hypermutated cancers. Strikingly, in MSS tumors, POLD1 mutations defy conventional immunotherapy paradigms by boosting immunogenicity through high TMB and clonal neoantigens, rendering these tumors responsive to ICIs. These insights elevate POLD1 as a potential predictive biomarker and a target for innovative approaches, such as neoantigen-based vaccines or synthetic lethal strategies exploiting replication defects. Moving forward, prospective studies must validate POLD1’s clinical utility, refine combinatorial treatments, and embed POLD1 profiling into routine practice. By decoding POLD1’s intricate roles, we pave the way for tailored therapies that could transform outcomes in cancers long resistant to immunotherapy, heralding a new era in precision oncology.
